# Anterolateral Bone Window for Revision Broken Cemented Stem of Unipolar Hemiarthroplasty

**DOI:** 10.1155/2021/6620395

**Published:** 2021-02-23

**Authors:** Mohamed Mosa Mohamed Mahmoud, Bahaaeldin Ibrahim, Amr Abdelhalem Amr, Maysara Abdelhalem Bayoumy

**Affiliations:** Orthopaedic and Traumatology Department, Faculty of Medicine in Assiut, Al-Azhar University, Assiut 71524, Egypt

## Abstract

**Background:**

Fractured stem of the hip prosthesis is well documented in the literature. Although it is rare, it is considered as a challenging problem. Many techniques have been described to solve this problem. *Purpose of the Study*. Evaluation of the effect of anterolateral bone window for extraction of the cemented femoral stem of hemiarthroplasty in revision total hip replacement.

**Methods:**

The study included eight revision hip arthroplasties in eight patients, with a broken stem of cemented (Thompson) hemiarthroplasty, which has been revised by the anterolateral proximal femoral window. All cases received cemented cups and cement-in-cement stems, except one case who received cementless long stem. Clinical follow-up of cases by Harries hip score (HHS) and X-ray.

**Results:**

Functional improvement of HHS of all cases, with no signs of loosening, after a mean follow-up period of 1.5 years.

**Conclusion:**

Extraction of broken stem is a challenging procedure. Many techniques have been described for revision of cases with a fractured stem of hip prosthesis, but we think that the anterolateral femoral bone window is a reproducible technique due to the characteristics of simplicity, short-time procedure, less invasive, not requiring extra instruments, and can be successful for most patients.

## 1. Introduction

Fractured stem of the hip prosthesis is well documented in the literature with an incidence rate between 0.23 and 11% [1, 2]. Three-dimensional analysis of stresses around the stem showed the highest concentration of stresses on the lateral aspect of the middle third, so fracture usually starts at the anterolateral aspect of the stem [3].

The causes of stem fracture can be categorized into patient factors: young and or overweight patients; technical factors: varus positioning of the stem or deficient cement; and implant factors: metallurgic deficiency [4, 5]. After the introduction of modern cementing techniques and the more resistant metal alloys, the incidence of stem fracture after hip replacement has been markedly reduced [1, 2]. However, there are some very few cases that may occasionally present with a broken stem especially with older designs that are used in the past such as cemented Thompson hemiarthroplasty, which is still used in some countries for fractured neck of femur in the older age group of patients. Removal of the distal part of a well-fixed broken cemented stem is a challenging procedure. Many techniques have been described, such as femoral bone window distal to the tip of the stem, extended trochanteric osteotomy, knee arthrotomy to push the distal part, and minimally invasive technique through the drilling of the proximal surface of the retained part of the stem and attachment of a threaded extraction device and a proximal femoral cortical window [6, 7]. In this series, we analyzed revision of the well-fixed broken stem of cemented Thompson hemiarthroplasty using anterolateral proximal femoral window without the need of extended trochanteric osteotomy.

## 2. Patients and Methods

**Figure 1:**
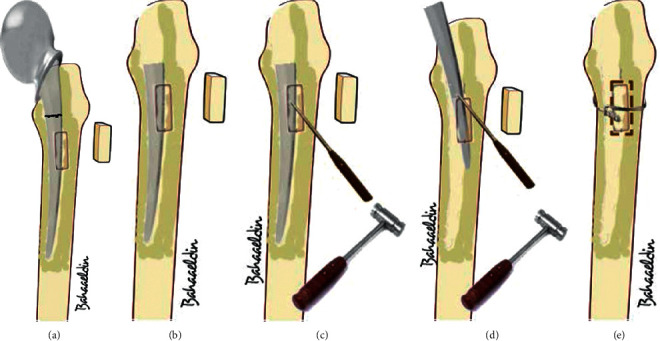
Sequence of events to extract fractured femoral stem. (a) Broken stem of Thompson prosthesis (b) rectangular bone window (1.5 cm width and 2.5 cm length) at the lateral aspect of the femoral shaft just distal to the lesser trochanter. (c) Small osteotome settled on the surface of the stem in an angle about 45° with the stem. (d) Hammering process to extract the distal part of the stem. (e) Cortical bone window is closed and secured with cerclage (diagrams were designed by Dr. Bahaaeldin Ibrahim).

**Figure 2:**
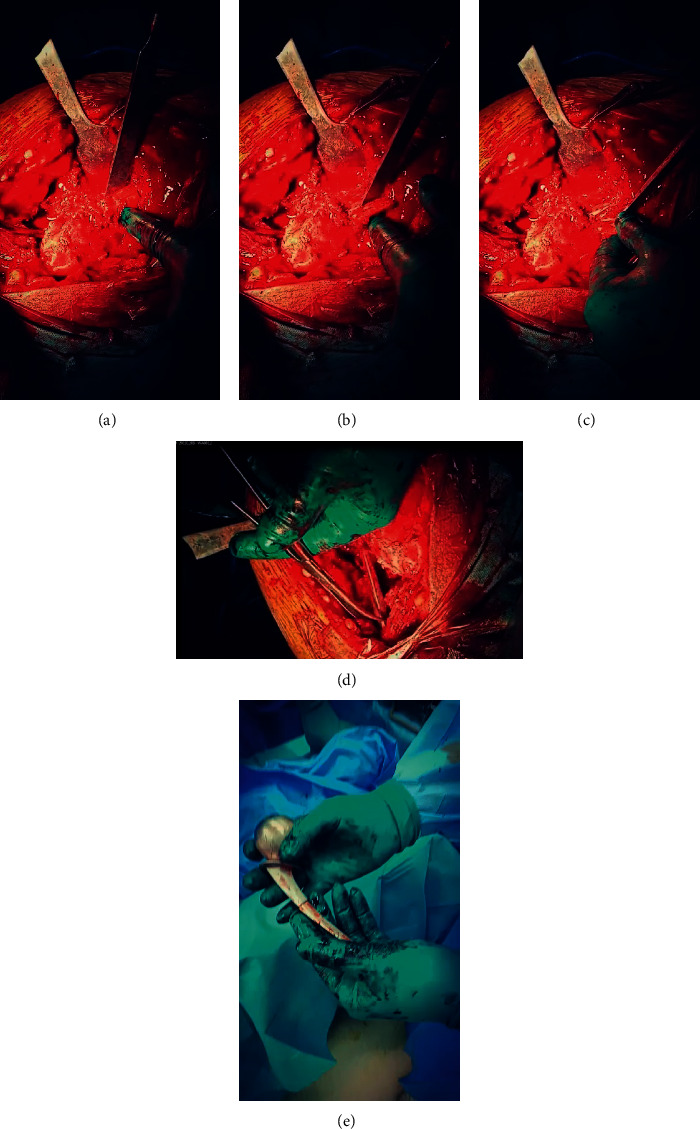
Intraoperative photos of window technique for stem removal. (a) A rectangular bone window done by saw at the lateral surface of proximal femur. (b) Elevation of the bone window to reveal the retained part of the stem. (c) Pushing the stem by an osteotome and hammer. (d) Delivery of the stem from the upper part of the femur. (e) Two parts of the Thompson prosthesis after removal.

**Figure 3:**
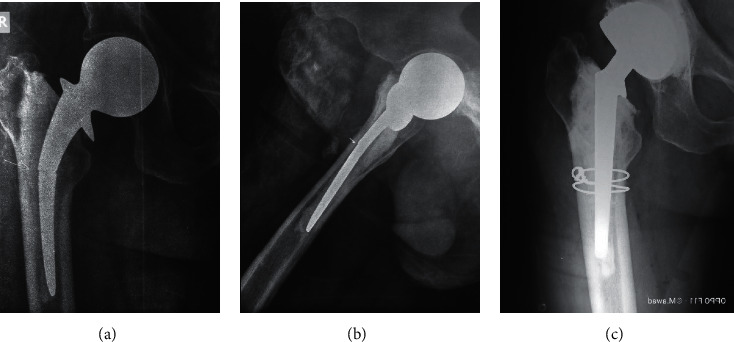
Preoperative and postoperative plane X-ray of a case with broken Thompson prosthesis. Removal of the broken stem by lateral bone window and revision by cemented dual mobility cup and DDH cemented stem.

**Figure 4:**
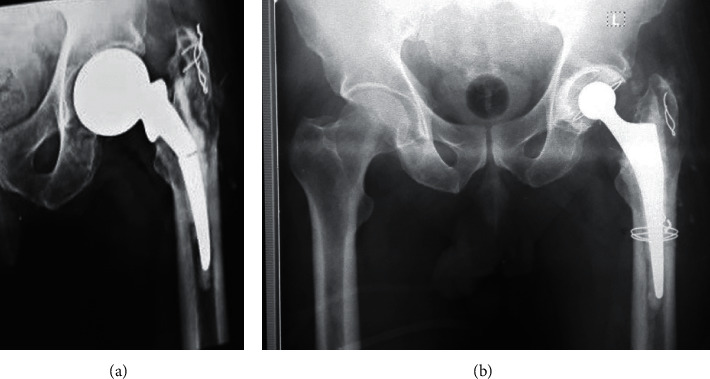
Preoperative and postoperative plane X-ray of a case with broken Thompson prosthesis. Removal of the broken stem by lateral bone window and revision by cemented polyethylene cup and cemented stem.

This retrospective study was approved by our Institutional Ethics Committee. From January 2017 till April 2019, eight revisions of hemiarthroplasty in eight patients have been done. The preoperative diagnosis of all cases was a broken stem of cemented (Thompson) hemiarthroplasty. The main indication for surgery was severe hip pain together with the inability to full weight bearing on the affected limb. The mean age of patients was 72 years old (range 65–76), and they were divided into two females and six males. The primary surgeries in all cases were done outside our hospital. Fracture of the stem in all cases occurred after minimal trauma. Stem fracture happened after a period of 1–5 years from the primary surgery. Preoperative ESR and CRP were done for all patients to exclude infection. There were no preoperative signs of infection in all cases. All cases underwent single-stage revision. Surgical technique (Figures 1(a)–1(e)): first, preoperative manual templating was done; lateral approach to the hip with patients on lateral decubitus position was done for all patients; removal of the proximal part of the prosthesis; and then removal of the superficial part of the cement mantle in the lateral aspect of the metaphyseal area of the femur. Then, a rectangular bone window (1.5 cm width and 2.5 cm length) had been done in the anterolateral aspect of the femoral shaft just distal to the lesser trochanter by an electric saw. This window was enough to expose the proximal end of the retained part of the stem. Then, small osteotome was settled on the surface of the stem in an angle about 45° with the stem. In two cases, we did two depressions on the surface of the stem by a carbide drill, connecting them to each other to make the osteotome settle on the stem. Hammering on the osteotome led to pushing of the distal part of the stem proximally until it was delivered from the femur upward. After that, the bone window was closed and secured by cerclage wires, and then cup implantation was done first (Figures 2(a)–2(e)). Cemented dual mobility metal cups were used in four patients (Figure 3(a)–3(c)) and cemented polyethylene cups in the other four patients (Figures 4(a) and 4(b)). Then, cement-in-cement conventional stems have been used in all cases except one case, where the stem was broken into three pieces and the window was extended distally, so cementless long stem has been used.

All patients started full weight bearing on the second postoperative day. Clinical assessment has been done by Harries hip score (HHS) after 3 months, 6 months, and then every year. A radiological examination was done in the postoperative day, 3 months, 6 months, and then every year. No intraoperative or postoperative complications have been detected.

## 3. Results

The mean follow-up period was about 1.5 years (from 1 year to 3 years). Functional outcome was markedly improved after surgery where the mean preoperative HHS was about 26 (25–30) and became 80 (65–85) postoperatively. Seven cases have good outcome, and one case has fair outcome. No signs of loosening in the cup or stem in the follow-up X-ray were seen. Union of the bone window has been detected in all cases. There were no intraoperative or postoperative complications.

## 4. Discussion

In this case series, we presented eight cases of revision cemented unipolar (Thompson) hemiarthroplasty after broken cemented stems of Thompson hemiarthroplasty, in which we used the anterolateral bone window techniques for extraction of the distal portion of stem. Most of the cases have good outcome. No signs of loosening in the cup or stem in the follow-up X-ray were seen. Union of the bone window has been detected in all cases. There were no intraoperative or postoperative complications. Mechanical failure of the prosthesis is one of the indications for revision THR. However, nowadays, it is of less common occurrence due to the development of more resistant metal alloys and the evolution of cementing techniques. The fractures of femoral stems have been previously described [8–12], and this type of failure may be due to excessive patient weight, high levels of physical activity, deficient bone support, malposition or loosening of the stem, the presence of a stress riser, and a reduced cross-sectional area within the stem [13–16]. In a study which included 122 patients using an extensively coated cobalt-chrome femoral component, Sotereanos et al. found two fractures of the stem (1.6 percent) [9]. Although Lakstein et al., in his study which included 72 hips at five to ten years of follow-up, reported only one stem fracture at the modular junction [12], Paprosky et al. found relatively high incidence which is six percent of femoral component fractures after revision hip arthroplasty. This may be due to reduction in the proximal support as a result of extended trochanteric osteotomy (ETO) against a distally well-fixed stem [8]. Collis used a technique in which he used a trephine for penetration of the cement-stem interface prior to extraction [5, 8]. Moreland et al. [17]. have described a technique that requires a femoral window through posterior cortex to expose the distal part of the broken femoral stem to perform retrograde impaction. Akwari et al. [7] described a modification of this technique in two patients, but performing reconstructions with standard stems and a cement-in-cement technique. The use of intramedullary nail for the retrograde impaction of the distal part of the stem through knee arthrotomy has also been described for selected cases [18]. The anterolateral bone window technique is easy applicable, less time-consuming than metal drilling, and better bone preservation compared to greater trochanter osteotomy. Although proximal femoral bone window technique has been previously published methods by Akwari et al. and Moreland et al. [7, 17], we did some modifications by making the bone window in the anterolateral aspect of the femur instead of posterior aspect because we did all cases by lateral approach and the anterolateral surface is more accessible than posterior, as we used the lateral approach to the hip in THA, we also used the electric saw instead of osteotome because it is much safer with less possibility in producing femoral fracture, and lastly, we used the cerclage wires instead of a metal cable which is cheaper. This technique is preferable to the distal window technique, where the bone window is done distal to the tip of the stem. Hence, a long-stem THR should be used to avoid stress rising and fracture of femur distal to the stem [17, 19]. But in the proximal window technique, we used a standard stem, which is less invasive, requires limited surgical time, and is cheap. In all of our cases, we did not use long-stem THA except in one patient, where the stem was broken to three pieces, so the window was slightly bigger and we have to remove the whole cement mantle and use cementless long-stem THA to gain better fixation distally and avoid fracture of the femur. The limitation of this study is the limited number of patients, but this is due to the rarity of incidence of fractured stem.

## 5. Conclusion

Extraction of broken stem is a challenging procedure, and many techniques have been described for revision of cases with fractured stem of hip prosthesis, but we think that the anterolateral femoral bone window is a reproducible technique due to the characteristics of simplicity, time sparing, less invasive, not requiring extra instruments, and can be successful for most patients.

## Data Availability

The methodology and patients' description are included in the article. All imaging data are available from the corresponding author on request. Personal details of patients' data are not allowed due to ethical concern.

## References

[1] Charnley J. (1975). Fracture of femoral prostheses in total hip replacement: a clinical study. *Clinical Orthopaedics and Related Research*.

[2] Heck D. A., Partridge C. M., Reuben J. D., Lanzer W. L., Lewis C. G., Keating E. M. (1995). Prosthetic component failures in hip arthroplasty surgery. *The Journal of Arthroplasty*.

[3] Hampton S. J., Andriacchi T. P., Galante J. O. (1980). Three dimensional stress analysis of the femoral stem of a total hip prosthesis. *Journal of Biomechanics*.

[4] Ritter M. A., Campbell E. D. (1986). An evaluation of Trapezoidal-28 femoral stem fractures. *Clinical Orthopaedics and Related Research*.

[5] Collis D. K. (1977). Femoral stem failure in total hip replacement. *The Journal of Bone and Joint Surgery*.

[6] Burgo F. J., Mengelle D. E., Feijoo M., Autorino C. M. (2019). A minimally invasive technique to remove broken cemented stems and its reconstruction with cement-in-cement. *HIP International*.

[7] Akrawi H., Magra M., Shetty A., Ng A. (2014). A modified technique to extract fractured femoral stem in revision total hip arthroplasty: a report of two cases. *International Journal of Surgery Case Reports*.

[8] Paprosky W. G., Weeden S. H., Bowling J. W. (2001). Component removal in revision total hip arthroplasty. *Clinical Orthopaedics and Related Research*.

[9] Sotereanos N. G., Engh C. A., Glassman A. H., Macalino G. E., Engh C. A. (1995). Cementless femoral components should be made from cobalt chrome. *Clinical Orthopaedics and Related Research*.

[10] Lakstein D., Eliaz N., Levi O. (2011). Fracture of cementless femoral stems at the mid-stem junction in modular revision hip arthroplasty systems. *The Journal of Bone and Joint Surgery*.

[11] Busch C. A., Charles M. N., Haydon C. M. (2005). Fractures of distally-fixed femoral stems after revision arthroplasty. *The Journal of Bone and Joint Surgery*.

[12] Lakstein D., Backstein D., Safir O., Kosashvili Y., Gross A. E. (2010). Revision total hip arthroplasty with a porous-coated modular stem: 5 to 10 years follow-up. *Clinical Orthopaedics and Related Research*.

[13] Andriacchi T. P., Galante J. O., Belytschko T. B., Hampton S. (1976). A stress analysis of the femoral stem in total hip prostheses. *The Journal of Bone and Joint Surger*.

[14] Buttaro M. A., Mayor M. B., Van Citters D., Piccaluga F. (2007). Fatigue fracture of a proximally modular, distally tapered fluted implant with diaphyseal fixation. *The Journal of Arthroplasty*.

[15] Miller E. H., Shastri R., Shih C. I. (1982). Fracture failure of a forged vitallium prosthesis: a case report. *The Journal of Bone and Joint Surgery: American Volume*.

[16] Ishaque B. A., Stürz H., Basad E. (2011). Fatigue fracture of a short stem hip replacement: a failure analysis with electron microscopy and review of the literature. *The Journal of Arthroplasty*.

[17] Moreland J. R., Marder R., Anspach W. E. (1986). The window technique for the removal of broken femoral stems in total hip replacement. *Clinical Orthopaedics and Related Research*.

[18] Szendroi M., Tóth K., Kiss J., Antal I., Skaliczki G. (2010). Retrograde genocephalic removal of fractured or immovable femoral stems in revision hip surgery. *HIP International*.

[19] Pellicci P. M., Salvati E. A., Robinson H. J. (1979). Mechanical failures in total hip replacement requiring reoperation. *The Journal of Bone and Joint Surgery*.

